# Probiotics and vitamins modulate the cecal microbiota of laying hens submitted to induced molting

**DOI:** 10.3389/fmicb.2023.1180838

**Published:** 2023-05-09

**Authors:** Chunyang Wang, Honghu Shan, Hui Chen, Xindong Bai, Jingru Ding, Dongyang Ye, Fathalrhman Eisa Addoma Adam, Yawei Yang, Juan Wang, Zengqi Yang

**Affiliations:** ^1^College of Veterinary Medicine, Northwest A&F University, Xianyang, Shanxi, China; ^2^Hongyan Molting Research Institute, Xianyang, Shanxi, China

**Keywords:** cecal microbiota, probiotic, vitamin, induced molting, laying hen

## Abstract

Induced molting enables laying hens to relax, restore energy and prolong the laying hen cycle, resolving problems such as poor egg quality and minimizing economic losses caused by rising global feeding costs. However, traditional molting methods may disrupt gut microflora and promote potential pathogens infections. This study used a customized additive with a mixture of probiotics and vitamins to induce molting and examine the cecal microbiota post molting. A total of two hundred 377 day-of-ISA Brown laying hens were randomly assigned to four groups: non-molt with basal diet (**C**), 12-day feeding restriction (FR) in earlier-molting (**B**), feed again to 27.12% egg production in middle-molting (**A**) and reach second peak of egg production over 81.36% in post-molting (**D**). Sequencing 16S rRNA to analyze cecal microbial composition revealed that there is no significant change in bacterial community abundance post-molting. In contrast to group C, the number of potentially harmful bacteria such as *E. coli* and *Enterococcus* was not found to increase in groups B, A, or D. This additive keeps cecal microbiota diversity and community richness steady. In cecal contents, hens in group **B** had lower *Lactobacillus, Lachnospiraceae and Prevotellaceae* (*vs***C**, **A**, and **D**), no significant differences were found between post-molting and the non-molting. Furthermore, cecal microbiota and other chemicals (antibodies, hormones, and enzymes, etc.) strongly affect immunological function and health. Most biochemical indicators are significantly positively correlated with *Prevotellaceae*, *Ruminococcaceae* and *Subdoligranulum*, while negatively with *Phascolarctobacterium* and *Desulfovibrio*. In conclusion, the additive of probiotics and vitamins improved the cecal microbiota composition, no increase in the associated pathogenic microbial community due to traditional molting methods, and enhances hepatic lipid metabolism and adaptive immunological function, supporting their application and induced molting technology in the poultry breeding industry.

## Introduction

Around 14 months of age, laying hens begin a 4-month natural molting process, which causes a decline in egg production and quality (Decuypere and Verheyen, 1986). Worldwide, laying hens’ lifespans and farmers’ incomes are improved through the practice of induced molting. Artificially induced molting is the most popular method, as it minimizes the need for feeding. This technique allows layers to cease production and molt intensively within 2 months, which improves production performance and egg quality, and prolongs the economic utilization period of laying hens. Hens have been rejuvenated *via* inducing molting around the world. Almost 75% of American layers use induced molting for flock replacement since the early 2000s (Holt, 2003). China began artificially molting laying chickens in the early 2000s. Most commercial egg producers started induced molting in early 2014 as egg prices climbed. Induced molting increases production, but gut health risks may follow. Intestinal microbiota is now considered a “second genome” due to developments in DNA sequencing technology. As we all know gut microflora regulates the health of animals and is associated with the development of various diseases (Spor et al., 2011). The impact of forced molting on the gut microflora has been of great interest.

It was discovered while investigating the fecal microbiota of old laying hens post molting via the fasting method that *Lactobacillus* was less and harmful bacteria like *E. coli* was more, hens are more susceptible to *Salmonella Enteritidis* (*SE*) (Holt, 2003; Han et al., 2019). Inducing molting with an alfalfa diet instead of fasting dramatically reduced *SE* colonization of cecal contents, liver, spleen, and ovaries in hens (Woodward et al., 2005). Low-calcium molting diets sustain intestinal lactic acid production to prevent *SE* colonization (Ricke et al., 2004). Furthermore, plant extracts such as portulaca olerace extract, chrysanthemum polysaccharides and resveratrol, etc. modulate the composition of the microbiota under pathological conditions, increasing the abundance of *Lactobacillus, and Bifidobacterium* in the cecum, while decreasing the colonization of opportunistic pathogens such as *E. coli, Enterococcus, and Prevotella* (Zhao X.H. et al., 2013; Tao et al., 2017). Supplemental resveratrol boosts short-chain fatty acid-producing bacteria while decreases LPS-producing bacteria. Reduced levels of *Bacteroides* and *Alistipes* and increased levels of *Lactobacillus* are all signs that resveratrol has helped reestablish a balance to the gut microbiota (Ding et al., 2021). Particularly, prebiotics like mannan oligosaccharide will also promote *L. acidophilus* colonization and inhibit the colonization of *SE*, *E. coli* and *Staphylococcus aureus* with therapeutic doses (Swapnil et al., 2021).

The Food and Drug Administration (FDA) and the Chinese Ministry of Agriculture both recognize the bacteria *Bacillus subtilis* (*B. subtilis*), *Lactobacillus acidophilus* (*L. acidophilus*), *Lactobacillus plantarum* (*L. plantarum*) and *Saccharomyces cerevisiae* (*S. cerevisiae*) as secure and efficient feed additives. Lowering cholesterol and increasing IgA, IgG, and IgM levels, respectively, are two ways in which *B. subtilis* and *L. acidophilus* decrease the risk of intestinal infections (Brisbin et al., 2011; Upadhaya et al., 2017; Wang et al., 2018). *B. subtilis* improves intestinal microecology and reduces *Salmonella* infection in broilers by boosting the growth of beneficial bacteria like lactic acid bacteria and inhibiting the colonization of harmful bacteria like *E. coli*. In addition, both *Lactobacillus*, *Acidophilus* and *Lactobacillus plantarum* also have the effect of inhibiting inflammation caused by *Salmonella* (Hillman et al., 1995; Penha Filho et al., 2015; Peng et al., 2016; Mazkour et al., 2019; Khochamit et al., 2020). Due to its strong intestine colonization, *S. cerevisiae* is employed in poultry breeding to promote beneficial bacteria and absorb harmful bacteria. *S. cerevisiae* reduces *SE* and *E. coli* and increases *Lactobacillus* in broiler intestines, which improves digestion and metabolism (Yalçın et al., 2013; Khalid et al., 2021; Siddik et al., 2022). Probiotics boost beneficial bacteria colonization and reduce harmful bacteria adherence to the intestinal epithelium to prevent illness.

Multivitamins have also been demonstrated to have a positive impact on intestinal microflora. Vitamin D supplementation alters the composition and richness of the intestinal microflora by decreasing *Veillonellaceae* and *Oscillospiraceae*, while increasing beneficial bacteria such *Bacteroidetes* and *Bifidobacteria* (Bellerba et al., 2021). Vitamin A dramatically raised Bacteroides/bacteroidetes ratio. Vitamin A deficiency increased *Enterococci*, while its addition increased *Lactobacillus* in mice’s intestines, affecting early viral infection (Lv et al., 2016; Liu et al., 2017). Vitamin C also helps the organism fight infection and eliminate risky microorganisms (Carr and Maggini, 2017). Vitamin D intake decreases *Veillonellaceae* and *Oscillospiraceae* and increases *Bacteroidetes* and *Bifidobacteria* in the gut microbiota (Singh et al., 2020). Vitamin E relieves stress and prevents *Salmonella enteritidis* infection in laying hens (Liu et al., 2019).

In addition, as is well known, the health status of an individual can be evaluated by a variety of specific biochemical indicators, and the occurrence of disease also leads to changes in the gut microflora. ALP and AST activities have been used as indicators to evaluate liver injury. Studies have shown that liver fibrosis and NASH lead to elevated serum AST and ALP, meanwhile that cecal microflora dysbiosis was associated with the severity of liver fibrosis and NASH (Hamid et al., 2019). TC, TG, LDL-C, HDL and cholesterol are biomarkers of blood lipid metabolism. Overnutrition reduces the transport efficiency of vLDL during liver fat deposition, increases the contents of LDL and plasma cholesterol, which affecting normal liver function and leading to the decline of egg production (Wu X.L. et al., 2021).

The latest study discovered that FLS alters cecal microflora with the relative abundance of *Collinsella*, *Turicibacter*, and *Enterococcus* decreased while *Bacteroides*, *Butyricicoccus*, and *Clostridium* increased (Liu et al., 2023). The gut microbiome during pregnancy and post-parturition was significantly altered in black rhino, which was also correlated with with progestagen concentration (Antwis et al., 2019). However when ovarian function disrupted, FSH and LH levels rise dramatically, whereas E2 levels decreased significantly, accompanied by an increase in *Bacteroidetes*, *Butyricimonas*, *Lachnobacterium*, and *Sutterella*, which these alterations of the gut microbiome were closely associated to the levels of FSH, LH, and E2 (Wu J. et al., 2021). Intestinal IgG and IgA levels are able to reflect individual’s pathogen resistance. Studies have shown that intestinal microbes increased immunoglobulin production, limited the colonization and adhesion of pathogens to the mucosa, and therefore protect against enteric infections (Sanidad et al., 2022). Therefore, assessing the potential of the cecal microbiome as a biomarker by studying the correlation between gut microflora and biochemical indicators will also provide a basis for early diagnosis and disease prevention in aged laying hens.

Probiotics and vitamins have been demonstrated to improve the gut microbiota of both broilers and laying hens, yet their application during the molting process has not been reported. In considering the previously mentioned benefits of probiotics and vitamins, we anticipated that administering this addition to induced-molting laying hens would have a similarly positive effect on microecological balance and the hens themselves. This experiment examines the effect of probiotics and vitamins on the diversity and composition of the cecal microflora in laying hens, as well as the correlation between the cecal microflora and various biochemical indicators, to provide a new additive option and theoretical support for the widespread use of induced molting.

## Materials and methods

### Laying hens and housing

200 ISA Brown laying hens, 377 days old, were purchased from a commercial farm in Weinan (Shanxi, China), which were randomly divided into four groups. Ladder cages held four chickens each (45 cm × 45 cm × 45 cm). Keep natural light.

### Dietary supplementation and experimental protocol

Normal feeding was provided for 7 days before the start of the experiment, and with the approval of the Northwest A&F University’s Animal Care and Utilization Committee. The proprietary molting technology of Hongyan was utilized in the induced molting procedures (Table 1).

**Table 1:** Flow table of the induced molting process.

Period	Items			
	Stage	Days	Feed	Water, light time
Implementation period	1–7	7	Stone powder, 50 g/hen	Water supply, 8 h
	8–9	2	Stone powder, 20 g/hen	Cut off water, 3 h
	10	1	Stop feeding	Resume water intermittently, 8 h
	11–14	4	Stop feeding	Water supply, 8 h
Recovery period	15	1	Breeding feed, 30 g/hen	Water supply, 8.5 h
	16	1	Breeding feed, 60 g//hen	Water supply, 9 h
	17	1	Breeding feed,75 g/hen	Water supply, 9.5 h
	18	1	Breeding feed, 90 g/hen	Water supply, 10 h
	19	1	Breeding feed, 110 g/hen	Water supply, 10.5 h
	20–23	4	Pre-laying feed, 120 g/hen	Water supply, 11 h
	24–29	6	Laying feed, 120 g/hen	Water supply, Daily increase, 0.5 h
	30–34	5	Laying feed, 120 g/hen	Water supply, daily increase to 16 h/d
2nd egg laying period	35–44	10	Laying feed,120 g/hen	Water supply, 16 h
	45–49	5	Laying feed, 120 g/hen	Water supply, 16 h
	49–143	94	Laying feed, 120 g/hen	Water supply, 16 h

One-way, fully randomized experimental design. Four groups: the control group (C) is non-molt with basal diet (Table 2), 12-day FR in earlier-molting (B), feed again to 27.12% egg production in middle-molting (A), and reach second peak of egg production over 81.36% in post-molting (D). Five chickens per treatment replicated 10 times. The three experimental groups began the molting operation in turn, collecting cecal samples at 425 days of age. Bacterial community changes in cecal contents of five samples from four groups were examined.

**Table 2:** Composition and nutrient contents of the basal diet.

Composition (%)	Breeding, pre-laying, and laying feed content (%)			Nutrient content	Breeding, pre-laying, and laying feed content (%)		
Corn	65.0	65.0	57.4	Crude protein, %	15.5	17.0	17.5
Soybean meal	16.4	16.4	29.0	AME, MJ/kg	2.8	2.75	2.7
Stone powder	6.0	6.0	3.81	Calcium, %	0.8	2.0	3.5
Wheat bran	7.0	7.0	3.175	Available phosphorus, %	0.60	0.55	0.60
Bone meal	1.5	1.5	2.5	Methionine, %	0.20	0.30	0.32
Shellfish powder	3.5	3.5	3.5	Lysine, %	0.45	0.60	0.65
Sodium chloride	0.3	0.3	0.37	Crude fibre, %	8.0	7.0	7.0
DL-Met	0.08	0.08	0.12	Crude Ash, %	10.0	13.0	15.0
Vitamin premix^a^	0.02	0.02	0.025	Moisture, %	12.0	12.0	12.0
Mineral premix^b^	0.1	0.1	0.1				
Total	100.00						

a Vitamin premix per kilogram contains: vitamin A 150,000 IU, vitamin D_3_ 20,000 IU, vitamin E 1,000 IU, vitamin K 55 mg, vitamin B_1_ 15 mg, vitamin B_2_ 8 mg, vitamin B_6_ 4 mg, vitamin B_12_ 0.4 mg, niacin 30 mg, pantothenic acid 0.55 mg, folic acid 0.4 mg, biotin 0.16 mg, choline chloride 450 mg.

b Provided per kilogram of diet: Cu, 12 mg; Zn, 100 mg; Fe, 120 mg; Mn, 50 mg; Se, 0.4 mg; I, 20 mg.

In the molting process, the specialized additive including *B. subtilis* (2.0 × 10^7^ cfu/mL), *L. acidophilus* (1.5 × 10^7^ cfu/mL), *L. plantarum* (1.0 × 10^7^ cfu/mL), *S. cerevisiae* (2.0 × 10^7^ cfu/mL) and multivitamins (Vitamin A 700,000 IU/L, Vitamin C 5,000 mg/L, Vitamin D 200,000 IU/L, Vitamin E 1,000 mg/L, Vitamin B_1_ 1,800 mg/kg, Vitamin B_2_ 3,000 mg/kg, Vitamin B_6_ 3,000 mg/kg) were added to drinking water and feed.

Aseptically collected 1.0 g cecal contents at four phases. All samples were flash-frozen in liquid nitrogen and stored at −80°C for further analysis. After 2 hours at 4°C, wing vein blood samples were kept overnight at 37°C. The serum then was centrifuged at 1,200×*g* for 10 min and kept at −20°C until analysis.

### Serum biochemical parameters

IgA, IgG, and IgM levels were measured by ELISA kits (BEIJING SINO-UK, China). The plasma biochemical indexes, including glutamic pyruvic transaminase (ALT), glutamic oxaloacetic transaminase (AST), total protein (TP), albumin (ALB), globulin (GLB), total cholesterol (TC), triglyceride (TG), alkaline phosphatase (ALP), low density lipoprotein (LDL) and high density lipoprotein (HDL), measured by the Mindray BS-420 automatic biochemical analyzer (Mindray company, China) and DR-200BS Microplate reader (HIWELL-DIATEK, China).Very low density lipoprotein (vLDL), estrogen (E_2_), progesterone (P), luteinizing hormone (LH), follicle stimulating hormone (FSH) and oxytocin(PRL) were all detected using ELISA kits (BEIJING SINO-UK, China).

### DNA extraction and 16 rRNA gene sequencing

Each sample’s DNA was extracted as previously mentioned. The NanoDrop2000 Spectrophotometer (Thermo Scientific Inc., United States) measured the extracted DNA’s concentration and purity, and agarose gel electrophoresis determined its integrity (Biowest, Spain).

Miseq sequencing the bacterial community. Create bacterial amplicon libraries using adapter primers 338F: (ACTCCTACGGGAGGCAGCAG) and 806R: (GGACTACHVGGGTWTCTAAT) for the V3-V4 regions of the 16S rRNA gene. After amplifying DNA with high-fidelity Taq polymerase (Invitrogen, USA), three equivalent PCR reactions per sample were mixed and then purified using the QIAquick PCR Purification Kit (Qiagen, Valencia, CA). The PCR settings were initial denaturation at 95°C for 3 min, 35 cycles consisting of 30 s at 95°C, 30 s at 55°C, and 45 s at 70°C, with a final extension by 10 min at 72°C. The AxyPrep DNA Gel Extraction Kit was used to purify the PCR results (Axygen Biosciences, Union City, CA, United States). Each sample involves three PCR rounds, then the three repeated PCR products were mixed and identified using 2% agarose gel electrophoresis. Using QuantiFluorTM Fluorescence, PCR products were detected, quantified, and mixed according to each sample’s sequencing needs. Sequencing was performed using an Illumina MiSeq PE300 platform (Majorbio Co. Ltd., Shanghai, China).

### Sequence analysis

Quality control of the original sequence was performed using Fastp software, and merged by FLASH software. After quality control splicing, the DADA2 plug-in in the Qiime2 process was used to minimize the noise of the optimized sequence using the default parameters. ASV (Amplicon Sequence Variant) encoding sequence and abundance data were obtained.

To reduce the impact of sequencing depth on alpha and beta diversity data analysis, 20,034 sample sequences were subsampled. Each sample’s Good’scoverage remained at 99.09%. The taxonomy of ASVs was examined using the Naivebayes classifier in Qiime2, which was based on the Sliva rRNA gene database (v 138). Mothur v1.30.1 calculated rarefaction curves and alpha diversity indicators from ASV data. ASVs, Chao1 richness and Shannon index were among these indicators.

### Statistical analysis

Majorbio Cloud analyzed the following data. Principal coordinate analysis (PCoA) was performed to compare and contrast the composition of the microbial communities in different samples. Discover bacterial taxa with substantial phylum-to-genus-level abundance differences using LEfSe analysis (LDA > 3.0, *p* < 0.05). Redundancy analysis (RDA) was used to select species for Spearman correlation network plot analysis to explore how clinical indicators affect the cecal bacterial population of laying hens.

## Results

### 16S rRNA sequencing information

The MiSeq platform was used for 16S rRNA gene sequencing. Following quality control, we obtained 1,018,542 readings with an average length of 418 bp for all samples. The number of 16S rRNA sequences retrieved ranged from 20,034 to 28,087 per sample. After subsampling the samples, a total of 2,398 bacterial ASVs were identified.

### Bacterial community richness and diversity

#### Bacterial community α-diversity

**Figure 1:**
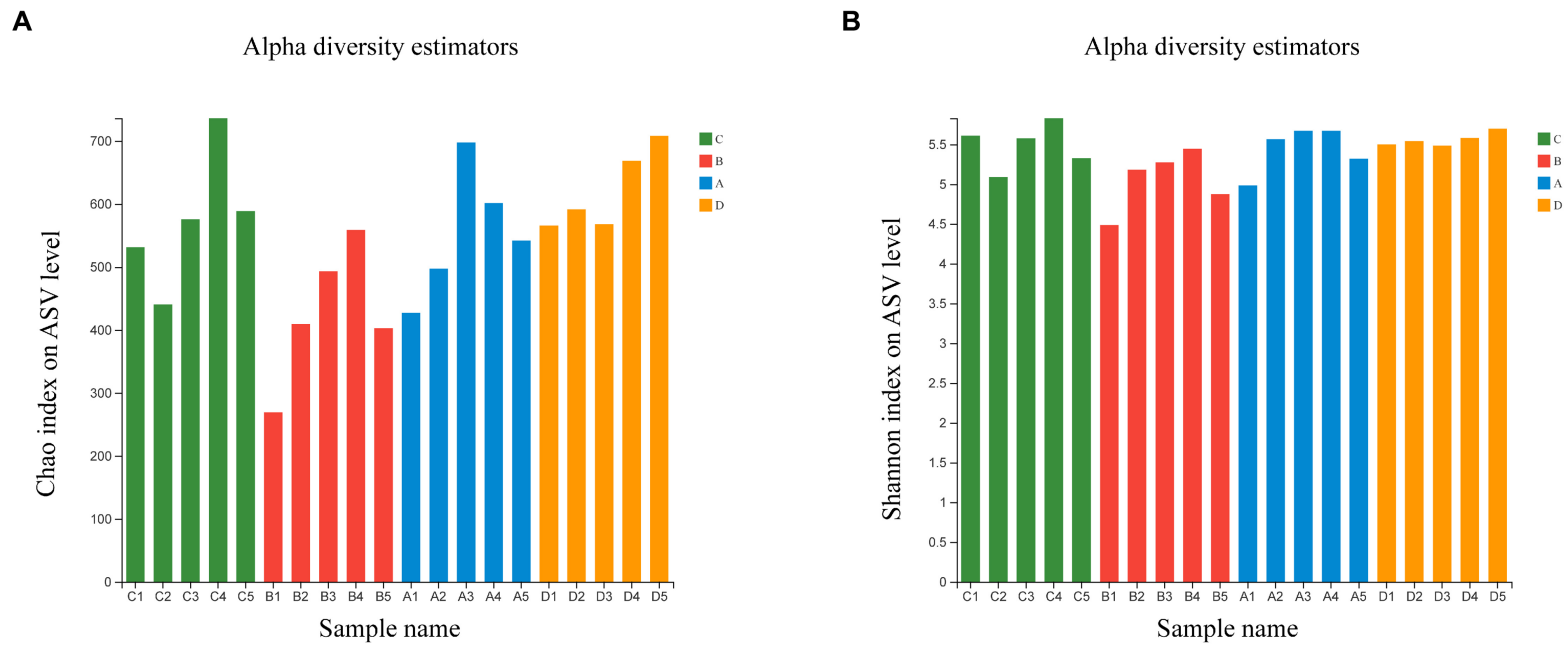
Estimated values of bacterial community relative abundances of Chao1 index **(A)** and Shannon diversity index **(B)**.

Comparing bacterial community relative abundances (Chao1) and diversity (Shannon) index values for each group (Figure 1). The Chao1 and Shannon index indicated that the bacterial community abundances and diversity in group B is less than those in group D (*p* < 0.05), other groups exhibited no statistical difference (Supplementary Figure S1). The results showed that FR could reduce the abundance and diversity of microflora, with the feeding resumption to the end of molting, the abundance and diversity of microflora gradually recovered and increased.

#### Bacterial community β-diversity

**Figure 2:**
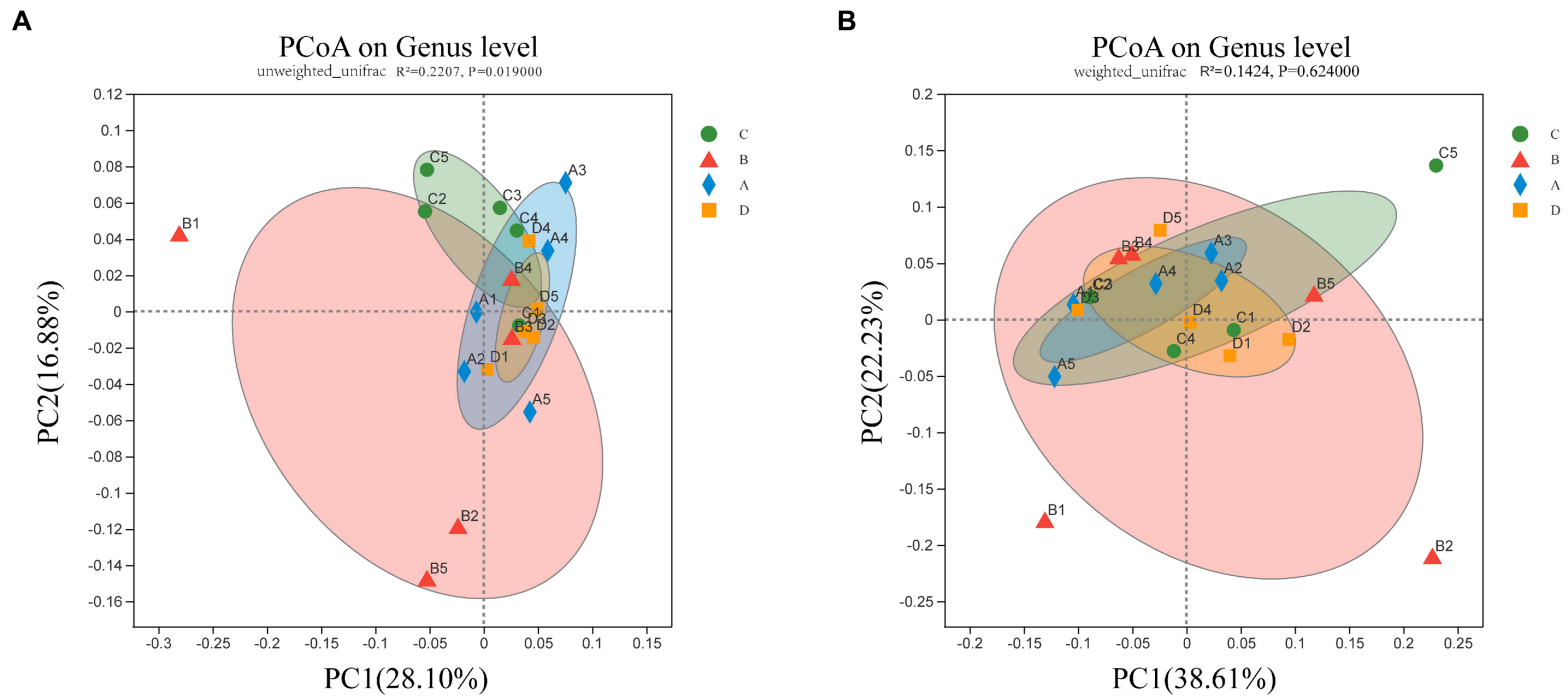
PCoA of bacterial in the cecal content. Bacterial community composition was calculated using **(A)** unweighted Unifrac (*p* = 0.019) and **(B)** weighted Unifrac (*p* = 0.624) across the groups C, B, A, and D (*n* = 5). The two axes (PC1 and PC2) explain 44.98% and 60.84% of the variation in bacterial species observed in cecal contents, respectively.

To address the differences in cecal microbial community composition between samples in different groups of molting, the PCoA based on Unweighted UniFrac distances shows that the group B separates from other groups, group C, A, and D tend to cluster together (Figure 2A) (*p* < 0.05). However, when β-diversity was assessed using Weighted UniFrac distances, which emphasize the seperation of group B, but without distinction among other three groups (Figure 2B) (*p* > 0.05). These findings showed significant changes of the cecal microbial communities in group B (FR), but prominent similarities of the microbial communities between the group C and group A and D in terms of the most abundant and dominant bacteria.

### Analysis of community species compositions

**Figure 3:**
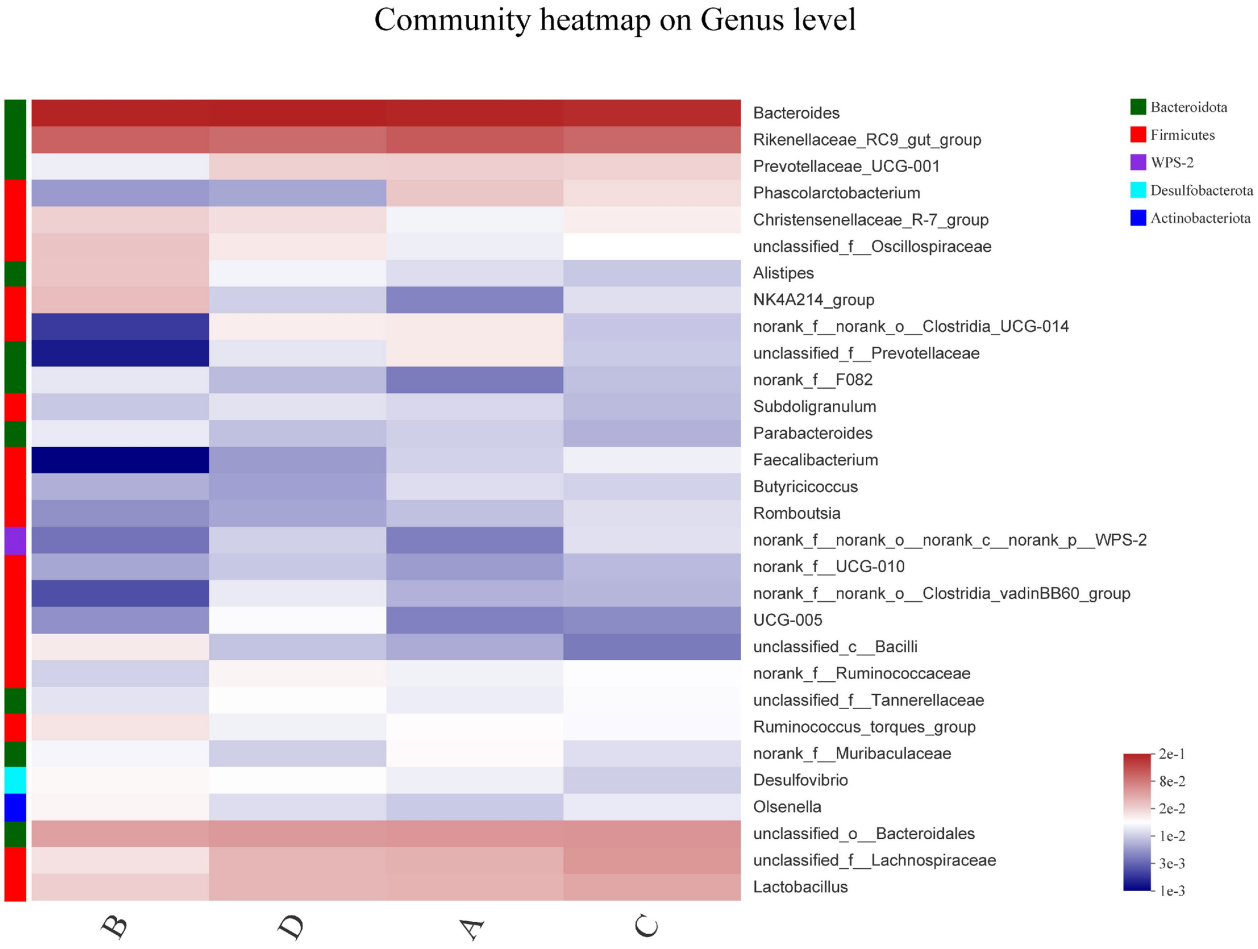
Bacterial community heatmap in cecal contents. The abundance of various species is indicated by the different color gradients in the two-dimensional structure. The horizontal axis represents the group, and the vertical axis represents one genus per row. The heatmap clusters high and low abundance species into blocks based on color variation and similarity, demonstrating similarity and variation between groups.

In order to explore the similarity and difference of community composition at the species level of the four groups, the heatmap plots was performed to cluster the top 30 species in terms of relative abundance in Figure 3. There were no significant changes in the dominant strains of *Bacteroides* and *Rikenellaceae*. FR reduced the relative abundance of *Prevotellaceae*, *Clostridia*, *Faecalibacterium* and *Lachnospiraceae*; and increased *Olsenella*, *Alistipes* and *Ruminococcus*. *Phascolarctobacterium* and *Faecalibacterium* decreased after 12 days of FR and post-molting, whereas *Oscillospiraceae*, *Alistipes*, *Bacilli* and *Desulfovibrio* increased. Overall, induced molting has an effect on the composition of cecal microflora.

### Differences in bacterial community composition

#### Bacterial composition at the phylum level

**Figure 4:**
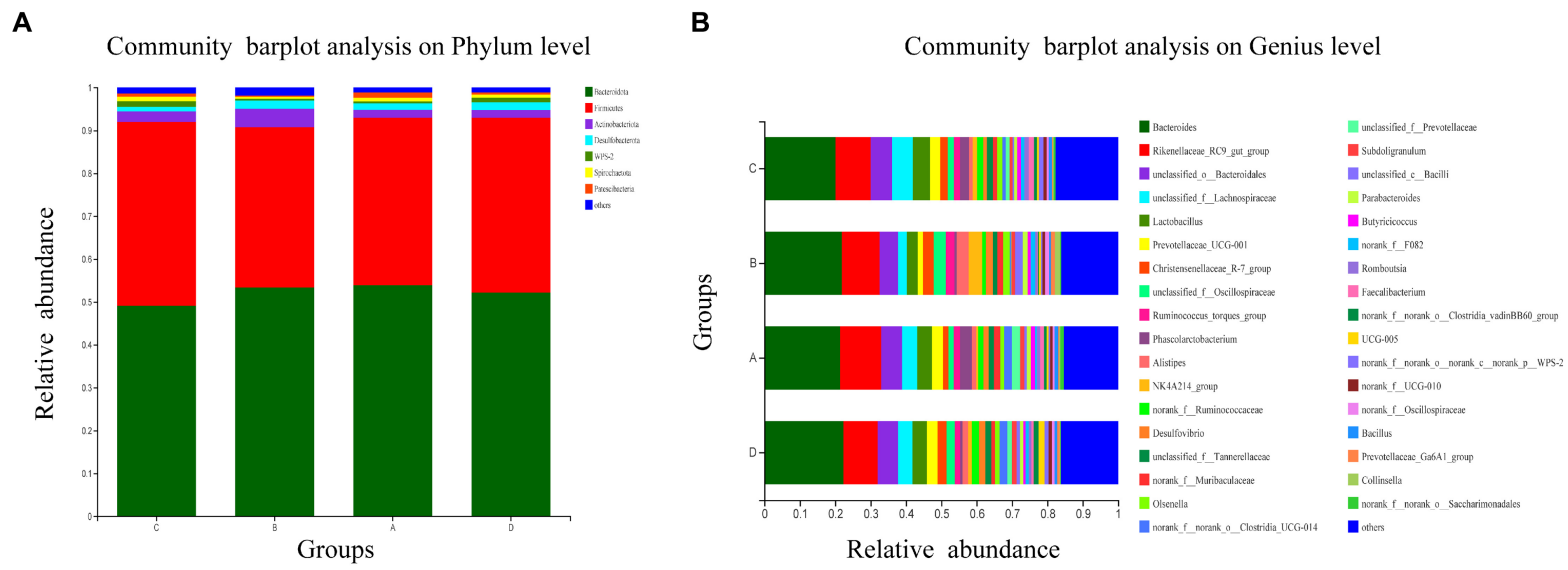
Taxonomic composition of the cecal microbiota at the phylum **(A)** and genus **(B)** level. Values are expressed as mean values. At the phylum and genus level, only taxa that represent more than 1% of the population in at least one group are listed.

ASV categorization is able to determine the exact percentages of each group at each level. At the phylum level, the *Bacteroides* and *Firmicutes* phyla were the most prevalent in the cecal (Figure 4A), accounting for more than 90% of total sequences. There was a slight increase in the amount of *Actinobacteria* in group B, but otherwise no significant change from groups A, C or D.

#### Bacterial composition at the genus level

The 30 most abundant genera are listed (Figure 4B). The dominant species in the bacterial community were generally consistent with the four groups, but species abundance varied. *Bacteroides*, *Rikenellaceae*, *Lactobacillus* and *Lachnospiraceae* dominated all samples.

Group B had the lowest relative abundance (percentage) of *Lactobacillus Lachnospiraceae* and *Prevotellaceae* in dominating species, and the most of *Christensenellaceae*, *Oscillospirceae* and *Alistipes*. However, groups C, A and D had similar bacterial abundances.

#### Bacterial composition difference at the specie level

**Figure 5:**
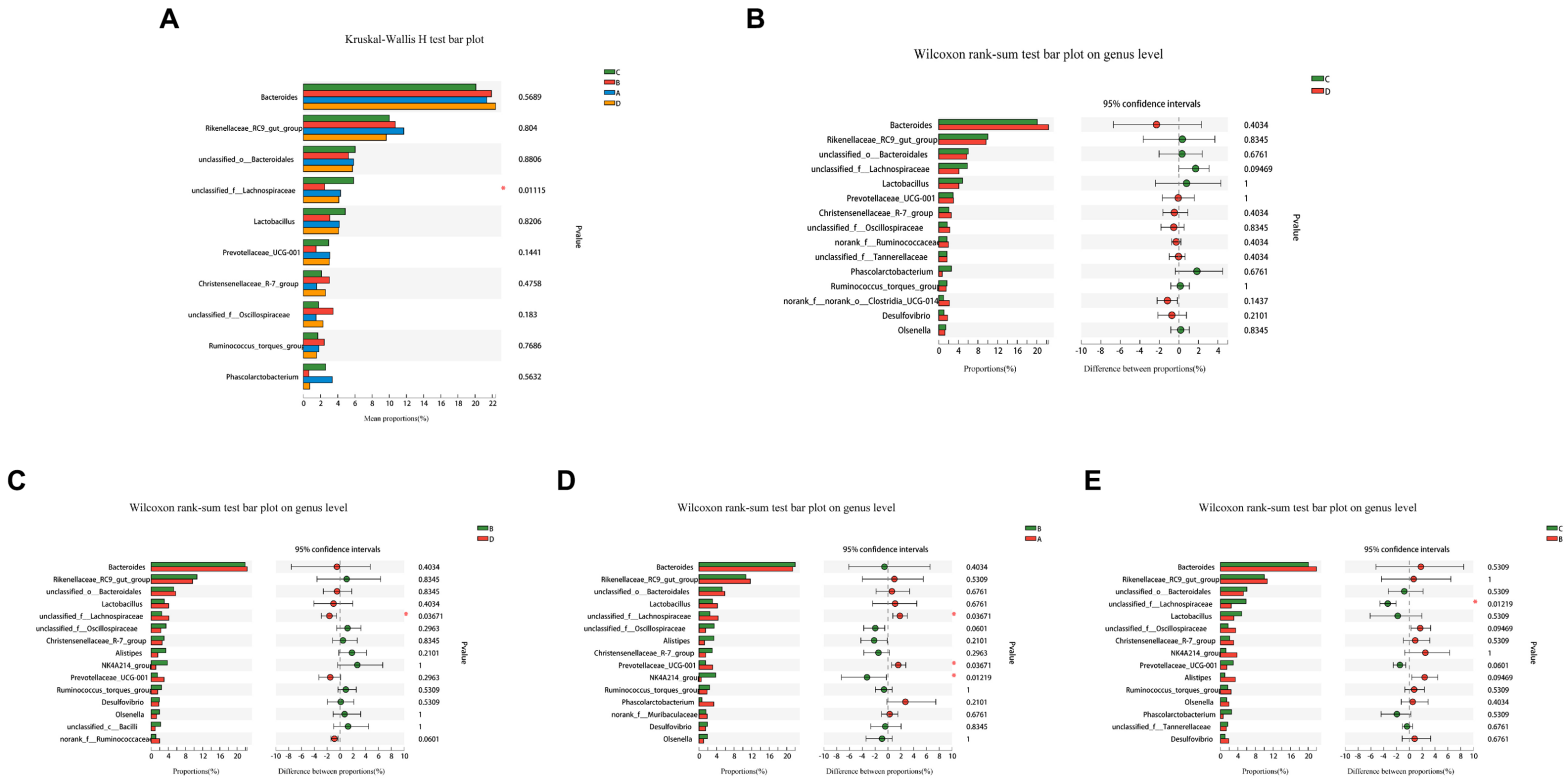
Analysis of variability in species abundance between groups. The Kruskal-Wallis H-test and the Wilcoxon rank sum test were used, respectively, and *p*-values were corrected using the “FDR” correction method. The use of an asterisk denotes statistically significant differences between groups. Comparison of the relative abundance of top 10 species in the four groups **(A)**, between-group variations C and D **(B)**, B and D **(C)**, B and A **(D)**, C and B **(E)**.

A comparative analysis of the abundance of the top 10 species in the four groups in multiple (Figure 5A) and a comparison of the two groups (Figures 5B–E) revealed that the abundance of *unclassified_f Lachnospiraceae* in group B was much lower than other three groups. Comparing the two groups, *Prevotellaceae_UCG-001* in group B was significantly lower than in group A, although the differences between other species were not statistically significant. FR causes temporary changes in the cecal microflora that recovers with the feeding resumption.

#### Differential microbiota compositions of LEfSe analysis

**Figure 6:**
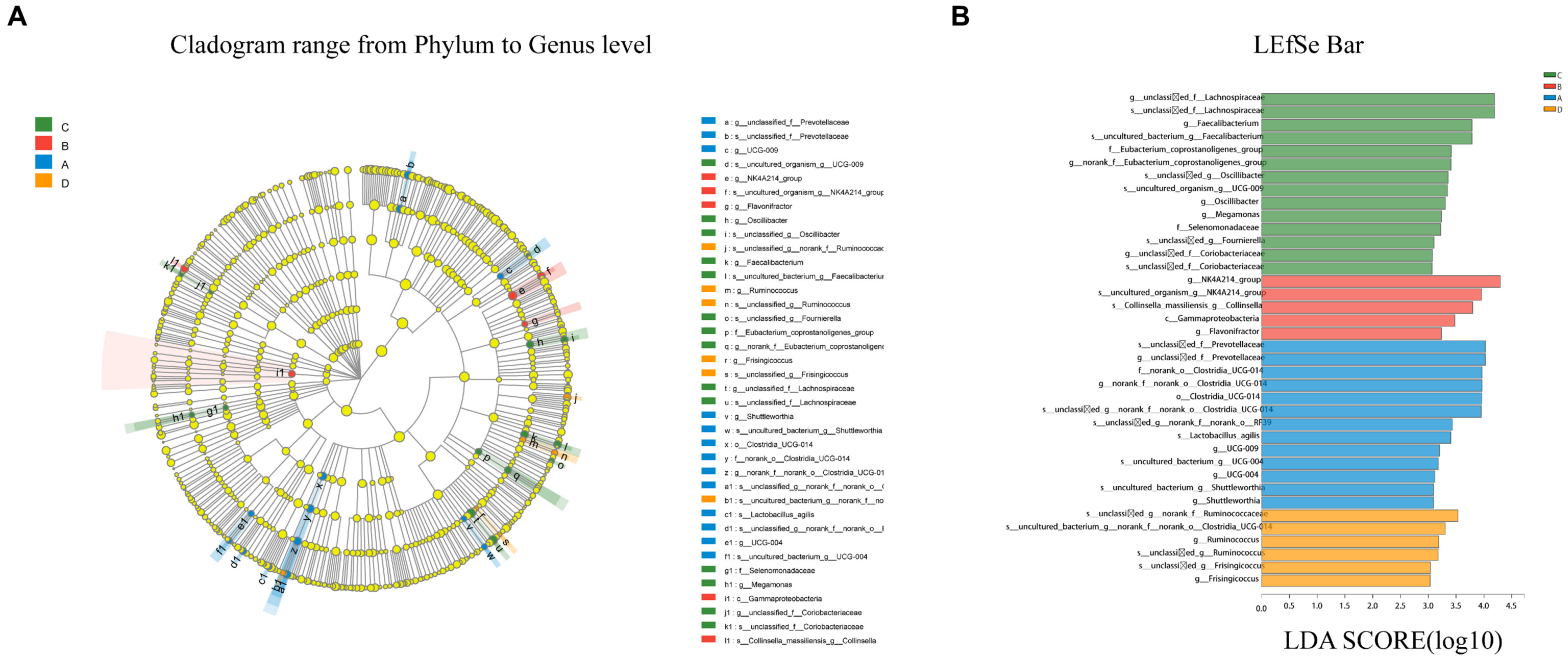
Cladogram reveals the phylogenetic distribution **(A)**. Linear discriminatory analyses (LEfSe) of four groups of bacterial taxa (LDA > 3.0) **(B)**. Different color nodes represent microbial taxa that are significantly enriched in the corresponding groups and influence group differences. Light yellow nodes represent microbial taxa that are not significantly different across groups. The abbreviations class (C), order (o), family (f), genus (g) and species are used (s). Longer bars indicate a greater influence of microbiota in the LefSe bar.

LEfSe multilevel species discrimination method was used to analyze the cecal microbiota. Although there was no statistically difference in β-diversity between the four groups (*p* > 0.05), species with different relative abundances were present as shown in the LEfSe analytical cladogram (Figure 6).

According to LEfSe, a total of 21 species differed in relative abundance between the four groups from the phylum to the genus level (LDA > 3.0, *p* < 0.05). LDA discriminant analysis reveals that *Clostridia* (from order to specie), *Prevotellaceae* (the genus and its specie), and *Shuttleworthia* (the genus and its specie) were significantly enriched in group A (*p* < 0.05). In group B, only *g_Flavonifractor*, *c_Gammaproteobacteria*, and *s_Collinsella* were detected to be significantly enriched (*p* < 0.05). In group C, six groups of bacteria were significantly enriched, namely *g_Oscillibacter* (the genus and its specie), *Eubacterium* (the family and its genus), *Faecalibacterium* (the genus and its specie), *Lachnospiraceae* (the genus and its specie), *Selenomonadaceae* (the genus and its specie), and *Coriobacteriaceae* (the genus and its specie). In group D, fewer microbes were significantly enriched, namely *Ruminococcus* (from genus to species) and *Frisingicoccus* (from genus to species).

### Correlation between the microbial community and serum biochemical indicators

Molting changes microbial community structures and immunity, reproductive hormones and metabolism characteristics. Measurements of immunity, reproductive hormones and heptic lipid metabolism-related indicators are shown in Tables 3–5, respectively. Blood levels of IgA, IgG and IgM were higher for group D than other groups. Among them, the levels of IgA and IgM post-molt were significantly higher than non-molt. Levels of P and E_2_ were higher for group D than other groups, FSH was lower than group C.

**Table 3:** Immunity-related indicators of laying hens in group C, B, A, and D^1^.

Items (g/L)	Group C	Group B	Group A	Group D
IgA	2.01 ± 0.09^b^	2.08 ± 0.06^b^	2.2 ± 0.08^a^	2.3 ± 0.06^a^
IgG	4.25 ± 0.14	4.22 ± 0.07	4.21 ± 0.09	4.3 ± 0.1
IgM	1.65 ± 0.06^b^	1.67 ± 0.04^b^	1.75 ± 0.09^b^	1.88 ± 0.09^a^

^1^Values are mean ± standard error. ^a,b,c^Means with no common superscripts differ significantly (*p* < 0.05) within a row. Different letters represent significant differences.

**Table 4:** Reproductive hormones-related indicators of laying hens in group C, B, A, and D^1^.

Items	Group C	Group B	Group A	Group D
P (ng/mL)	0.71 ± 0.01^b^	0.72 ± 0.01^b^	0.73 ± 0.02^b^	0.76 ± 0.02^a^
E_2_ (pg/mL)	21.23 ± 3.33^b^	28.21 ± 13.96^b^	24.81 ± 2.77^b^	43.15 ± 4.53^a^
LH (mIU/mL)	16.86 ± 5.01	14.08 ± 3.92	14.6 ± 2.17	14.9 ± 1.75
FSH (mIU/mL)	5.66 ± 1.05^a^	4.9 ± 0.48^ab^	5.5 ± 0.09^ab^	4.68 ± 0.25^b^
PRL (μIU/mL)	253.72 ± 11.17	254.13 ± 18.4	258.68 ± 7.9	259.5 ± 33.34

^1^Values are mean ± standard error. ^a,b,c^Means with no common superscripts differ significantly (*p* < 0.05) within a row. Different letters represent significant differences.

**Table 5:** Heptic lipid metabolism-related indicators of laying hens in group C, B, A, and D^1^.

Items	Group C	Group B	Group A	Group D
AST (U/L)	129.4 ± 13.53^b^	102.14 ± 14.38^c^	129.32 ± 10.41^b^	159.76 ± 13.02^a^
ALT (U/L)	59.72 ± 18.5^a^	26.04 ± 0.13^b^	30.4 ± 2.19^b^	36.57 ± 4.47^b^
ALP (U/L)	433.49 ± 143.34	283.66 ± 128.05	270.58 ± 133.17	323.85 ± 68.33
TP (g/L)	51.11 ± 4.65^a^	22.92 ± 3.78^b^	46.33 ± 13.27^a^	57.43 ± 3.26^a^
ALB (g/L)	18.49 ± 3.33^a^	7.74 ± 1.28^b^	16.27 ± 5.93^a^	19.29 ± 2.29^a^
TC (mmol/L)	2.78 ± 0.56^a^	1.29 ± 0.1^b^	2.73 ± 0.99^a^	2.82 ± 0.18^a^
TG (mmol/L)	13.16 ± 1.47	12.17 ± 0.86	11.49 ± 0.87	11.32 ± 1.56
LDL-C (mmol/L)	0.96 ± 0.1^b^	0.74 ± 0.11^c^	1.11 ± 0.21^ab^	1.21 ± 0.09^a^
HDL-C (mmol/L)	1.39 ± 0.33^a^	0.56 ± 0.1^b^	1.24 ± 0.66^ab^	1.78 ± 0.58^a^
vLDL (mmol/L)	0.94 ± 0.09^ab^	0.75 ± 0.14^b^	1.05 ± 0.24^a^	1.16 ± 0.1^a^

^1^Values are mean ± standard error. ^a,b,c^Means with no common superscripts differ significantly (*p* < 0.05) within a row. Different letters represent significant differences.

Levels of AST, ALT, TP, ALB, TC, LDL-C, and HDL-C indicated a decreasing and then increasing trend, these indicators were much lower for group B than group C. Compared to group C, ALT significantly decreased, LDL-C and vLDL-C greatly increased for group D. Overall, the moulting process is associated with changes in antibodies, hormones, and other indicators, indicating alterations in immunity, physiological regulation, and lipid metabolism.

**Figure 7:**
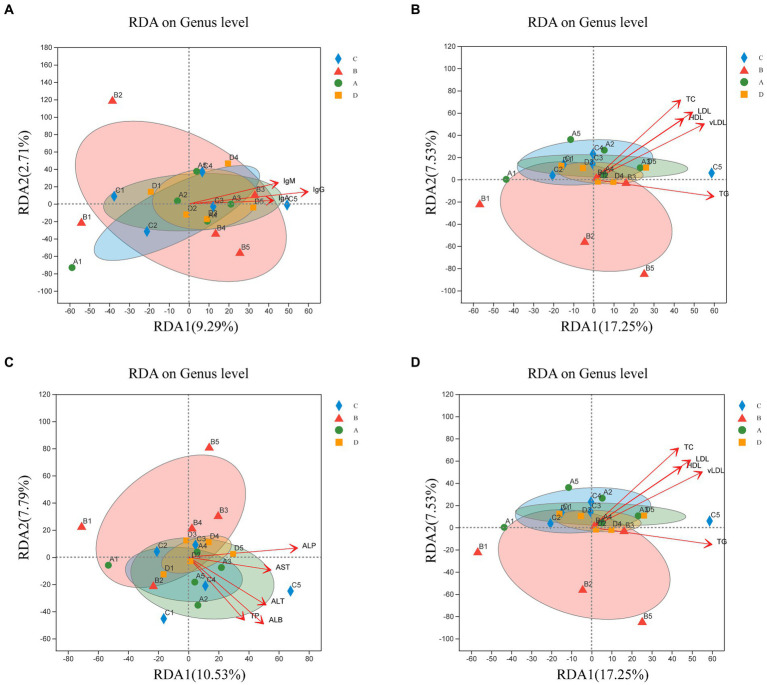
Redundancy analysis (RDA) of sample distribution (shapes) and serum biochemical indicators (arrows). Correlation of the microbial community with IgA, IgG, and IgM **(A)**; P, E_2_, LH, FSH, and PRL **(B)**; AST, ALT, ALP, TP, ALB, and GLB **(C)**; TC, TG, HDL, LDL, and vLDL **(D)**. The values of axes 1 and 2 indicate the percentages depicted by the corresponding axes, reflecting the degree of correlation.

Therefore, this study investigated whether microbial community structure and immunity, reproductive hormones and lipid metabolism characteristics are related. The RDA investigated the link between serum biochemical indicators and microbial communities (Figure 7). Microbial community composition correlated positively with IgG (*p* = 0.003), IgM (*p* = 0.043), E_2_ (*p* = 0.023), LH (*p* = 0.005), FSH (*p* = 0.023), PRL (*p* = 0.046), ALT (*p* = 0.034), ALP (*p* = 0.005), TP (*p* = 0.039), ALB (*p* = 0.003), TC (*p* = 0.001), TG (*p* = 0.026), HDL (*p* = 0.01), LDL (*p* = 0.002) and vLDL (*p* = 0.003).

**Figure 8:**
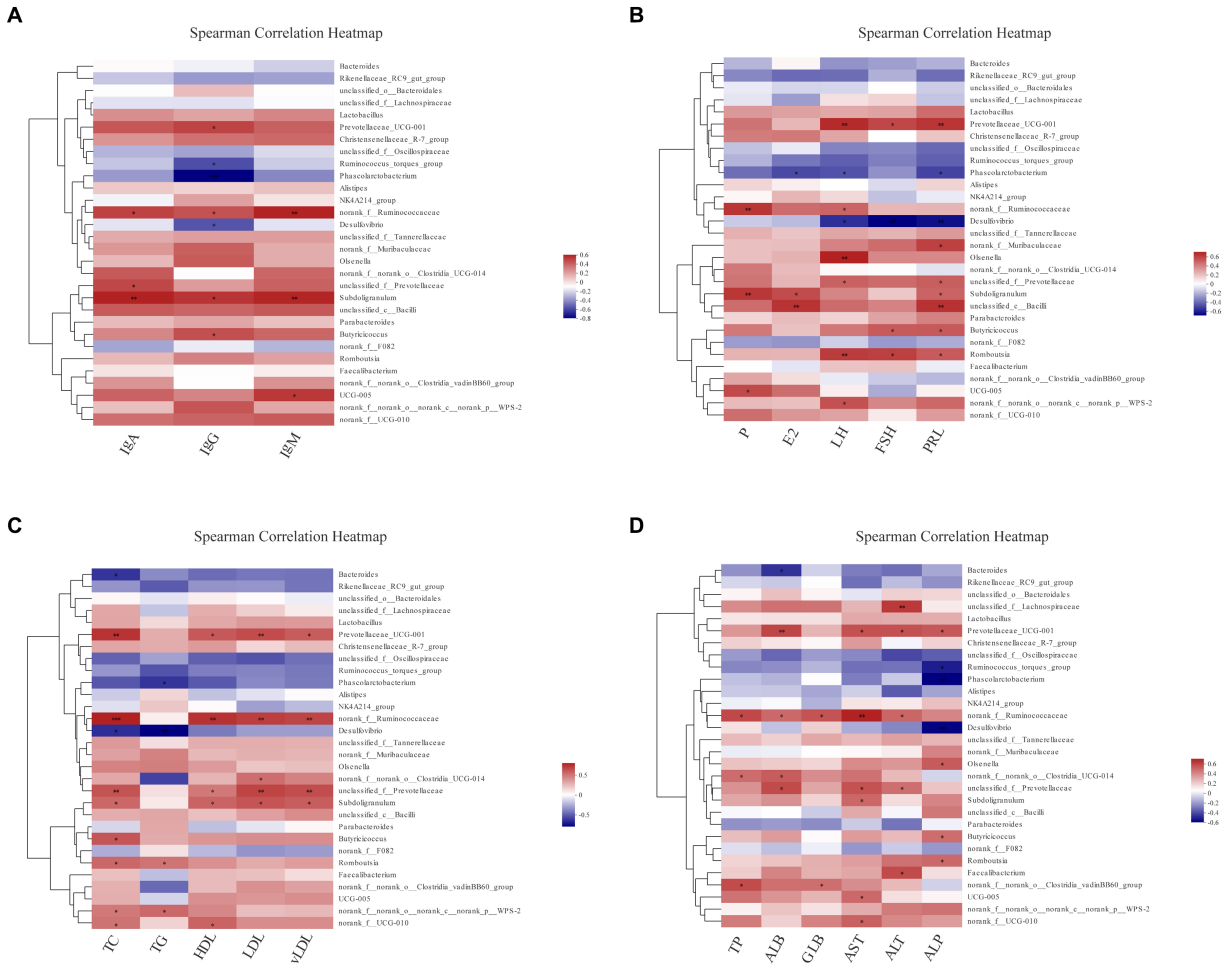
The analysis of Spearman’s rank correlation between biochemical indicators and cecal microbiotas. The correlation coefficients are colored, and the right color card of the heatmap is a color partition of different values (*p* < 0.05, 0.01, 0.001 is marked as ∗, ∗∗, ∗∗∗ in black color, respectively). Spearman’s correlation analysis of cecal microbiota with IgA, IgG and IgM levels **(A)**; with P, E_2_, LH, FSH, and PRL levels **(B)**; with TC, TG, HDL, LDL and vLDL levels **(C)**; with TP, ALB, GLB, AST, ALT and ALP levels **(D)**.

To completely analyze the relationship between immunity, reproductive hormones and lipid metabolism-related indicators, and cecal-associated microbiota, the Spearman correlation coefficient was calculated to generate the correlation matrix. As shown in Figure 8, we observed that most biochemical indicators (IgA, IgG, IgM, TC, HDL, LDL, vLDL, and AST) were positively correlated with *Prevotellaceae UCG-001*, *norank_f__Ruminococcaceae*, *Subdoligranulum* (*p* < 0.05), *Phascolarctobacterium* and *Desulfovibrio* were negatively correlated with IgG, LH, FSH, PRL, TG, TC, ALP, etc. (*p* < 0.05). *Prevotellaceae UCG-001* and *Romboutsia* were positively correlated with LH, FSH, and PRL (*p* < 0.05). *Furthermore, Bacteroides, Rikenellaceae_RC9_gut_group* and u*nclassified_f__Oscillospiraceae* were all negatively but not significantly correlated. Cumulatively, our results suggest that cecal microbiota was associated with reproductive hormones, lipid metabolism-related indicators and immunoglobulins during the molt.

## Discussion

### No significant changes in microbial diversity and abundance post-molting

Short laying cycle and poor egg quality have always been the main problems worldwide in laying hens in the late phase. It is well known that forced molting can prolong egg production cycle and improve production performance, but the traditional forced molting method will cause intestinal microflora disturbance and promote potential pathogen infection. Therefore, the effect of the new induced molt method of laying hens on the cecal microflora in this experiment is worthy of exploration. Intestinal microecology imbalance caused by traditional forced molting stress may increase the incidence of bacterial infections (Han et al., 2019). First, we use the new induced molt method with the additive of probiotics and vitamins to induce molting and revealed that FR reduced the abundance and diversity of microflora; with the feeding resumption, the abundance and diversity of microflora post-molting gradually increased, and there was no significant difference from that of the non-molting group.

In current experiment, Bacteroidetes and Firmicutes, two major bacterial phyla, accounted for over 90% of the cecal microbes of molting hens, confirming prior findings (Oakley et al., 2014; Han et al., 2019), although the abundance of *Firmicutes* (nearly 40% on average) lower than the data (47.5%) reported before (Qi et al., 2019).

The abundance of *Bacteroidetes* was higher than the reported value (11%) (Zhao L. et al., 2013). FR for 12 days (Group B) increased the abundance of the phyla *Actinobacteria* (4.25%) (*p* < 0.05), accompanied by weight loss (Pallotto et al., 2018). *Actinobacteria* may be therefore negatively associated with feed intake. Furthermore, the abundance of *Actinobacteria* (the phylum includes *Bifidobacterium*) and *Akkermansia* was associated with vitamin A addition (Huda et al., 2019). In this study, *Actinobacteria* quickly revert to normal levels with the feed intake and the addition of vitamin A, minimizing FR-induced potential microbiome imbalance.

At the genus level, *Bacteroides* (22.38% relative abundance), *Rikenellaceae_RC9_gut_group* (11.69% relative abundance) predominated the cecal microbiome, similar to previous research (Artdita et al., 2021). As the current study demonstrated, *Unclassified_o_Bacteroides*, *Lactobacillus* and *unclassified Lachnospiraceae* were also more prevalent in the cecal microbiome of laying hens (Sergeant et al., 2014).

### The additive of probiotics and vitamins balance FR-induced microflora

In general, feed intake level has a considerable effect on nutrient digestion, which may reduce host feed efficiency and perhaps alter the gut microbiota composition (Ortigues and Doreau, 1995). Therefore, any dietary supplement applied to improve hen’s performance will alter the gut microbiota, thereby altering the microbe-host interactions.

In group B, unweighted PCoA reveals a community composition change (*p* < 0.05) and only the u*nclassified_f__Lachnospiraceae* had a significant decline in abundance for the top 15 species. Thus, FR altered bacterial community diversity, but there was no significant difference in bacterial community composition after resumimg feeding post-molting and non-molting, with feeding resumption and the addition of probiotics and vitamins. *Lachnospiraceae* levels were higher in non-alcoholic fatty liver patients and may contribute to fatty liver progression (Shen et al., 2017). Whereas in this study, *Lachnospiraceae* decreased, suggesting FR may benefit to fatty liver.

Studies have found that the abundance of *lactobacilli* in excreta bacterial community decreased post-molting of laying hens (Han et al., 2019). In our study, there was no difference in the abundance of *lactobacilli* post-molting compared with non-molted laying hens. It may be related to the probiotics and vitamins used in the present study. It has been reported that the *L. plantarum* significantly increased the amount of *Lactobacillus* on fecal microbial composition (Qiao et al., 2019). The number of *Lactobacillus* spp. and *Bifidobacterium* spp. increased in diets supplemented with *B. subtilis* and *L. acidophilus* (Chen et al., 2022).

*S. cerevisiae* supplementation improves intestinal barrier function overall by increasing the number of lactic acid bacteria in the feces (Yamabhai et al., 2016), significantly raising IgG, reducing the presence of *SE* and attenuating *E. coli*-induced intestinal damage (Mountzouris et al., 2015; Wang et al., 2016; Sampath et al., 2021).

*B. subtilis* significantly reduced the minimum viable count of *E. coli*, *coliforms* and *staphylococci in the intestinal of laying hens,* while *L. acidophilus* reduced the number of *Clostridium* spp. (Forte et al., 2016). Adding *B. subtilis* alone did not improve the amount of probiotics in rabbits (Phuoc and Jamikorn, 2017), implying that we should focus on the combined utilization of multiple probiotics in the poultry farming process. The interaction of probiotics, including *Lactobacillus*, *Lactobacillus acidophilus*, *Bifidobacterium* and *S. cerevisiae*, will boost the number of beneficial bacteria and reduce the amount of potentially harmful or pathogenic bacteria such *E. coli*, *Enterococcus*, *Streptococcus*, and *Clostridium*. This trial also found no rise in potential pathogens bacteria post-molting, which is believed to be closely related to the addition of multiple probiotics, and these findings support the application of multiple probiotics during molting in laying hens.

Vitamin supplementation promotes intestinal beneficial bacteria, which improves the performance and quality of egg production in aged hens (Gan et al., 2020a). Several studies report that vitamin A and D deficiency alters the microbiota diversity and *Firmicutes*/*Bacteroides* ratio, thereby affecting host immunity and metabolic (Chen et al., 2020; Singh et al., 2022). Evidence has demonstrated that Vitamin C addition enhances the shape of intestinal villi, boosts the diversity and richness of the microorganisms in the cecal, and reduces *Bacteroidetes/Firmicutes* ratio and *Enterobacteriaceae*. The intake of vitamin C significantly raises the amount of enteric sIgA in laying hens and lessens *SE* damage to broilers (Gan et al., 2020b). The current findings are the first to indicate that adding a variety of probiotics and vitamins during molting prevented cecal microbiota imbalance.

Probiotics and vitamins improved the composition of the gut microbiota while inhibiting the growth of potentially harmful bacteria such as *SE* and *E. coli* during the molting. Hence, novel additives may help balance cecal microflora and improve intestinal health in aged laying hens post-molting.

### Correlation between bacterial community structure with immunity, reproductive hormones and hepatic lipid metabolism

#### With immunity

Intestinal immunity is crucial for preventing illness and maintaining overall health (Dupont et al., 2020). The three most abundant immunoglobulins are IgM, IgA, and IgG, which serve as early defense, mucosal immune and continuous antigen attack, respectively.

Brown algae was reported to improve serum IgG concentrations in rats, as well as *Prevotella* and *Subdoligranulum* abundance (Kim et al., 2018). Spearman’s rank correlation analysis in this trial showed that *Prevotella*, *Ruminococcaceae* and *Subdoligranulum* were all significantly and positively correlated with IgG and IgA. Dietary supplementation with *L. acidophilus* and *B. subtilis* increase the levels of IgG and IgA and the diversity of gut microbiota in laying hens (Chen et al., 2022). In addition, adding *S. cerevisiae* to the goat diet increased the relative abundance of *Ruminococcus* with significantly raised IgG and IgA level (Emu et al., 2021), suggesting that these probiotic strains may increase immunoglobulin levels by altering the composition of the gut microflora, which in turn may improve immune function.

#### With reproductive hormones

The metabolism, immunity and behavior of the host are all impacted by the microbes and hormones (Qi et al., 2021). Changes in the gut microbiota affects ovarian follicle and oocyte maturation, which is closely related to FSH and LH (Franasiak and Scott, 2015). Additionally, oestrogen also changes intestinal microbiota (Plottel and Blaser, 2011). Further investigations have demonstrated that alterations in gut microflora are associated with considerably higher serum LH and FSH levels in postmenopausal women and that certain probiotic supplements reduce serum LH and increase serum FSH (Chakravarti et al., 1976; Li et al., 2022). *Prevotellaceae UCG-001* and *Romboutsia* are strongly associated with LH, FSH and PRL (Figure 8), nevertheless, further investigation is needed to ascertain the relationship between various microflora and reproductive hormones. Future research may focus on early intervention using special microflora to prevent health problems associated with hormonal changes, whether in laying hen or human.

#### With hepatic lipid metabolism

Gut and liver are the two main organs involved in lipid and lipoprotein metabolism. Abnormalities in lipid metabolism may be caused by decreased liver function with elevated TG or LDL-C. Gut microflora can modulate lipid and lipoprotein metabolism, thereby reducing blood lipoprotein abnormalities (Yu et al., 2019). Some reports suggested that serum biochemical indicators of hepatic lipid metabolism are linked to gut microbiota changes. Firstly, *Ruminococcus_gauvreauii_group*, *Lachnoclostridium, Blautia, Allobaculum* and *Holdemanella* were significantly positive correlated with TG, TC, and LDL (Tang et al., 2018). In rats, *L. plantarum* lowered TG, TC, and LDL while increasing lipid levels. TG, TC, and LDL were positively correlated with *Blautia*, *Clostridium*, and *Roseburia* (Li et al., 2020). These data confirm the role of gut microbiota’s role in lipid metabolism.

Secondly, *Eubacterium coprostanoligenes* have been proved to improve dyslipidemia by influencing TC, HDL, and body weight (Wei et al., 2021). According to the Heatmap results (Figure 8), *Bacteroides*, *Rikenellaceae_RC9_gut_group* and *unclassified_f__Oscillospiraceae* were negative correlation with TC, HDL, LDL, and vLDL. This study lays the groundwork for further research on the role of gut microbiota in lipid reduction in laying hens.

Hepatic steatosis suddenly decreases egg output and quality, and fatty liver haemorrhage syndrome (FLHS) kills thousands commercial laying hens (Liu et al., 2022). *Coriobacteriaceae* was discovered to be relatively common in NAFLH and associated illnesses, with significantly higher AST levels (Tamura et al., 2021). *Firmicuts* and *Actinbacteria* were positively correlated with AST and ALP (Hamid et al., 2019). In our research, ALB, AST, and ALT were positively correlated with *Prevotellaceae_UCG-001*, *norank_f__Ruminococcaceae* and *unclassified_f__Prevotellaceae*. Overall, these findings point to a correlation between the gut microbiota and biomarkers associated with clinical liver disease. However, it is too early to confirm how the gut microbiota of hens be modified by external interventions to reduce the risk of liver disease. Further investigations are required to ascertain the role of particular microorganisms play in improving lipid metabolism in layers to counteract performance losses in the late laying phase.

## Conclusion

The additive of probiotics and vitamins administered during induced molting improves the diversity and composition of the cecal microecology, prevents microecological imbalance and increase in conditioned pathogenic bacteria caused by starvation-forced molting, and enhances immunity, reproductive hormone secretion and hepatic lipid metabolism post-molting. We believe that these findings will contribute to the widespread application of probiotic and vitamin additives in the molting of laying hens.

## Data availability statement

The datasets presented in this study can be found in online repositories. The names of the repository/repositories and accession number(s) can be found in the article/Supplementary material.

## Author contributions

CW was responsible for experimental design, data analysis, and manuscript writing. XB, HS, and JD contributed to sample collection. FA, HC, and DY provided to writing review. YY contributed to materials and methods support. JW and ZY contributed to writing review, editing, and project administration. All authors contributed to the article and approved the submitted version.

## Conflict of interest

The authors declare that the research was conducted in the absence of any commercial or financial relationships that could be construed as a potential conflict of interest.

## Publisher’s note

All claims expressed in this article are solely those of the authors and do not necessarily represent those of their affiliated organizations, or those of the publisher, the editors and the reviewers. Any product that may be evaluated in this article, or claim that may be made by its manufacturer, is not guaranteed or endorsed by the publisher.
