# Supplementary figures and images for: Probiotics and vitamins modulate the cecal microbiota of laying hens submitted to induced molting
Source: Front Microbiol. 2023 May 9;14:1180838. doi: 10.3389/fmicb.2023.1180838 (PMC10203222; doi:10.3389/fmicb.2023.1180838)

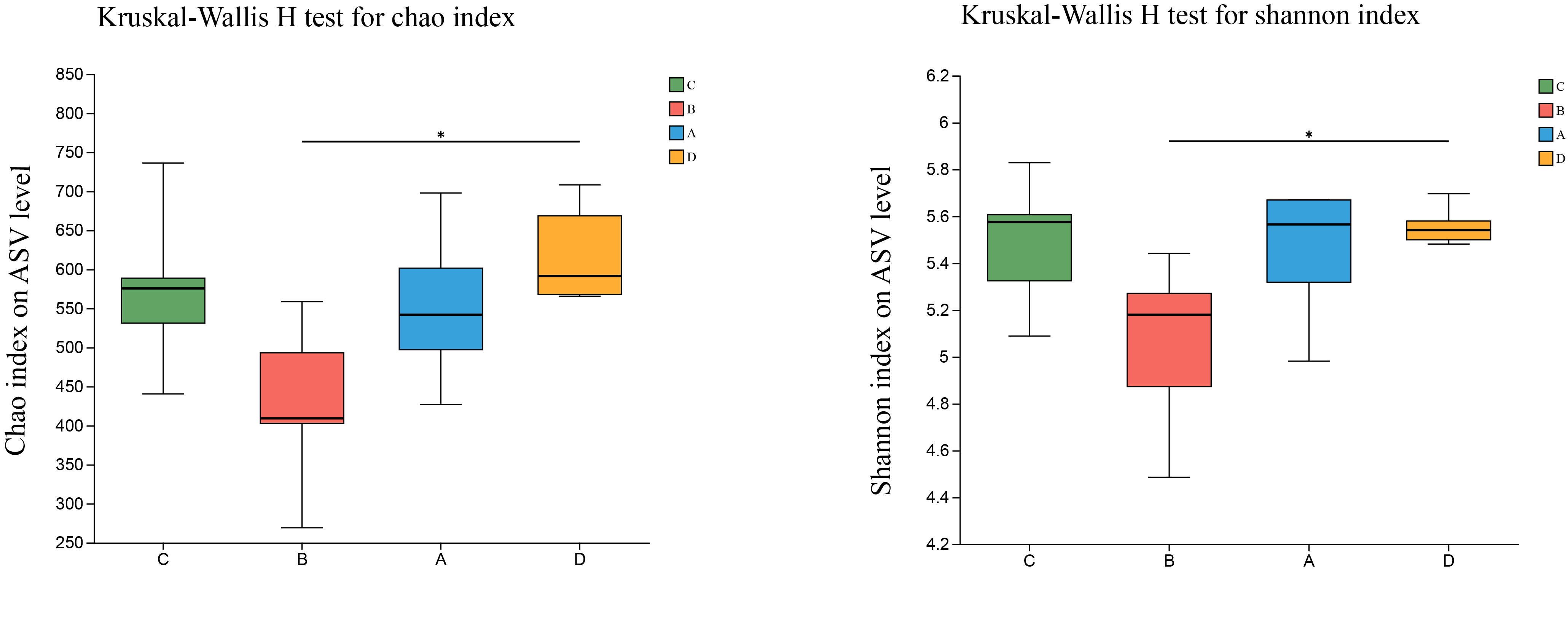

Supplement: Supplementary file 1 [file Image_1.PNG]
